# Analytical and numerical quantification and comparison of the local electric field in the tissue for different electrode configurations

**DOI:** 10.1186/1475-925X-6-37

**Published:** 2007-10-15

**Authors:** Selma Čorović, Mojca Pavlin, Damijan Miklavčič

**Affiliations:** 1 University of Ljubljana, Faculty of Electrical Engineering, Ljubljana, Slovenia

## Abstract

**Background:**

Electrochemotherapy and gene electrotransfer are novel promising treatments employing locally applied high electric pulses to introduce chemotherapeutic drugs into tumor cells or genes into target cells based on the cell membrane electroporation. The main focus of this paper was to calculate analytically and numerically local electric field distribution inside the treated tissue in two dimensional (2D) models for different plate and needle electrode configurations and to compare the local electric field distribution to parameter *U/d*, which is widely used in electrochemotherapy and gene electrotransfer studies. We demonstrate the importance of evaluating the local electric field distribution in electrochemotherapy and gene electrotransfer.

**Methods:**

We analytically and numerically analyze electric field distribution based on 2D models for electrodes and electrode configurations which are most widely used in electrochemotherapy and gene electrotransfer. Analytical calculations were performed by solving the Laplace equation and numerical calculations by means of finite element method in two dimensions.

**Results:**

We determine the minimal and maximal *E *inside the target tissue as well as the maximal *E *over the entire treated tissue for the given electrode configurations. By comparing the local electric field distribution calculated for different electrode configurations to the ratio *U/d*, we show that the parameter *U/d *can differ significantly from the actual calculated values of the local electric field inside the treated tissue. By calculating the needed voltage to obtain *E *> *U/d *inside the target tissue, we showed that better electric field distribution can be obtained by increasing the number and changing the arrangement of the electrodes.

**Conclusion:**

Based on our analytical and numerical models of the local electric field distribution we show that the applied voltage, configuration of the electrodes and electrode position need to be chosen specifically for each individual case, and that numerical modeling can be used to optimize the appropriate electrode configuration and adequate voltage. Using numerical models we further calculate the needed voltage for a specific electrode configuration to achieve adequate *E *inside the target tissue while minimizing damages of the surrounding tissue. We present also analytical solutions, which provide a convenient, rapid, but approximate method for a pre-analysis of electric field distribution in treated tissue.

## Background

Electroporation, also termed electropermeabilization, is a phenomenon where increased permeability of cells exposed to an external electric field is observed. The induced transmembrane voltage presumably leads to the formation of aqueous pores in the phospholipid bilayer, which increases the permeability of the cell membrane for water-soluble molecules and ions [1-4]. Electropermeabilization is currently widely used in vivo and in vitro in many biological and medical applications including electrochemotherapy of tumors (ECT) [5-7], transdermal drug delivery [8,9] and gene electrotransfer [5,10-14].

Electropermeabilization is a phenomenon, where the membrane becomes permeable after the magnitude of the electric field (*E*) exceeds reversible threshold value (*E*_*rev*_), while *E *below *E*_*rev *_does not significantly affect the cell membrane. When the magnitude of local electric field *E *reaches irreversible threshold value (*E*_*irrev*_), electric field causes permanent damages on the cell membrane leading to cell death. The threshold values, *E*_*rev *_and *E*_*irrev *_vary for different tissues in range from 200–400 V/cm and 450–900 V/cm, respectively [15-19]. Electropermeabilization with *E *in the range of *E*_*rev *_≤ *E *<*E*_*irrev *_reversibly permeabilizes the cell membrane and at the same time does not affect the viability of a biological cell. Reversible electropermeabilization has been proven to be successful in electrochemotherapy, where electric field enables chemotherapeutic drug to enter into tumor cells, and for gene electrotransfer, which can be used for gene therapy, where electric field enables DNA to enter the target cells. Irreversible electroporation with *E *> *E*_*irrev *_was suggested for water treatment and food preservation as a method for destruction of the cell membrane of noxious microorganisms and for tissue ablation [20-22].

In this paper we focus on the importance of calculating the local electric field distribution for successful electrochemotherapy tumor treatment and gene electrotransfer of target cells. Namely, for successful electrochemotherapy it is crucial that all clonogenic cells forming tumor tissue are exposed to the local electric field above the threshold value *E*_*rev *_and preferably below irreversible threshold *E*_*irrev*_. Similarly, successful gene electrotransfer also requires local electric field in the range of reversible electroporation regime (*E*_*rev *_≤ *E *<*E*_*irrev*_). It was previously shown by combining numerical modeling and experimental approaches that the efficacy of the electrochemotherapy and gene electrotransfer treatment depends on the magnitude of the local electric field inside the target tissue [17,18,23-28].

However, both threshold values (*E*_*rev*_, *E*_*irrev*_) differ for electrochemotherapy and gene electrotransfer as well as they depend on pulse parameters and the type of treated tissue.

From the theoretical principles it follows that the local electric field inside the tissue is in general a function of time and place *E*(*x*, *y*, *z*, *t*). However, since most often electric pulses used in electrochemotherapy and gene electrotransfer are usually long (0.1 – 10 ms) compared to the typical constant for the polarization of the cell membrane (around 1 μs), we can assume steady-state conditions for our analysis [29,30]. The local electric field distribution *E*(*x*, *y*, *z*) in the tissue is a complex function of several parameters. It depends on the applied voltage on the electrodes, the geometry and position of the electrodes, and on the non-homogeneous properties and geometry of the tissue. For this reason electric field distribution during electroporation can not be solved analytically except for the most simple cases [31] and therefore numerical methods have to be used [16,25].

In principle there are two complementary approaches to determine the optimal electrode configuration and applied voltage to achieve appropriate local electric field inside the target tissue (*E *≥ *E*_*rev*_). Ideally one should calculate *E *for each individual case taking into account all geometric details and electric properties of the treated tissue in order to assure appropriate local *E *inside the target tissue (i.e. pretreatment planning). However, this requires sophisticated numerical modeling for each individual problem and is in many cases not realistic. Alternatively some approximate estimates of *E *inside the target tissue are used, where usually a gross approximation *U/d *"electric field intensity" as defined and reported in a number of different reports [8-10,12,15,32-36] is used as an approximate value of *E *for plate as well as for needle electrodes.

Most of the experimental and clinical studies on electrochemotherapy were performed with the treatment protocol (applying eight 100 μs long pulses at the repetition frequency 1 Hz) using the parameter (*U/d*) from 1300–1500 V/cm to select applied voltage on the electrodes [26,37]. However, despite the fact that the parameter *U/d *is widely used in order to determine the applied voltage, this parameter alone does not give the information about the actual electric field inside the target tissue. It also makes difficult the comparison between different studies reported, especially since exact geometry is usually not given.

In this study we present an approach of local electric field evaluation, by means of 2D numerical and analytical models which can be used to determine the appropriate electrodes and electrode configurations and applied voltage in electrochemotherapy and studies of gene electrotransfer. We numerically and analytically compare *E*(*x,y*) in 2D for different electrodes and electrode configurations which are used for in vivo electrochemotherapy and gene electrotransfer. We demonstrate that the calculated local electric field inside the target tissue strongly depends on the chosen electrodes and electrode configuration and can be significantly different than the value *U/d*. In order to quantify and compare different electrode configurations we visualized the regions inside the treated tissue exposed to the local electric field exceeding the value *U/d *(*E *≥ *U/d*) keeping the value *U/d *for all configurations constant so that the electric field distribution can be directly compared between electrode configurations. In addition, we calculate the necessary voltage for a given electrode configuration in order to achieve adequate electric field distribution in the target tissue. We also demonstrated that changing electrodes' orientation and electrode arrangement with respect to the target tissue leads to better exposure of the target tissue to the adequate electric field distribution.

## Methods

### Numerical calculations

**Figure 1:**
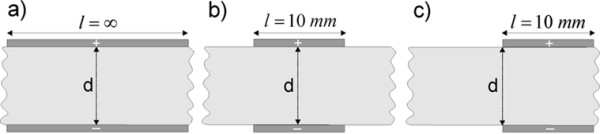
Three geometries with parallel plate electrodes analyzed in this study (d = 8.66 mm).

**Figure 2:**
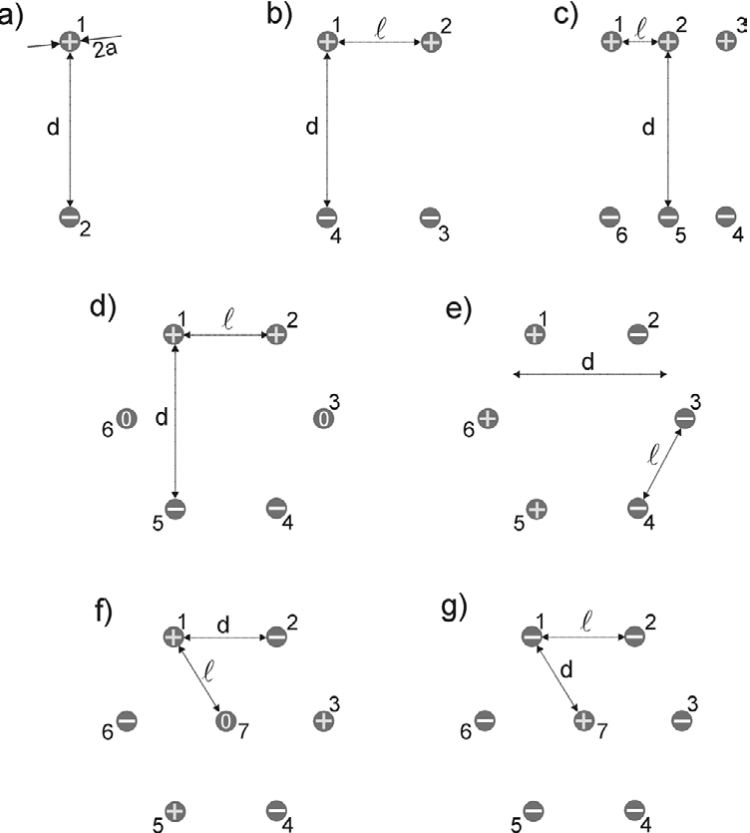
Different needle electrode configurations analyzed in this study.

Numerical calculations were performed by means of finite element method (FEM) [38] using FEMLAB software packages Femlab 2.3 and 3.0 (Comsol, Sweden). The numerical calculations were performed on the personal computer Intel Pentium 4, 2.40 GHz CPU and 1 GB RAM. The electric field distribution in 2D models was calculated using the steady current module. We analyzed *E*(*x,y*) for two parallel plate electrodes (Fig. 1) and different number (2, 4, 6 and 7) and configurations of needle electrodes as shown in Fig. 2. These configurations were chosen based on different reports [15,25,26,33,39-41] where such electrodes and electrode configurations were used in electrochemotherapy and gene electrotransfer in vivo experiments.

In all models the electrodes were positioned inside a square representing homogeneous tissue having a constant conductivity. A constant voltage was assigned to the grid points in regions where electrodes were placed, while insulation boundary conditions were set on the remaining boundaries. In all cases the constant voltage was applied between the electrodes giving *U/d *= 1.15 V/cm. The radius *a *of all needle electrodes was 0.215 mm. The distance *d*, defined as the distance between the positive and the negative electrode, was d=53 mm
 MathType@MTEF@5@5@+=feaafiart1ev1aaatCvAUfKttLearuWrP9MDH5MBPbIqV92AaeXatLxBI9gBaebbnrfifHhDYfgasaacH8akY=wiFfYdH8Gipec8Eeeu0xXdbba9frFj0=OqFfea0dXdd9vqai=hGuQ8kuc9pgc9s8qqaq=dirpe0xb9q8qiLsFr0=vr0=vr0dc8meaabaqaciaacaGaaeqabaqabeGadaaakeaacqWGKbazcqGH9aqpcqaI1aqndaGcaaqaaiabiodaZaWcbeaakiabbccaGiabb2gaTjabb2gaTbaa@349D@ for configurations shown in Figs. 2a–2e, whereas in configurations shown in Figs. 2f and 2g we set *d *= *l *= *5 mm*. The dimension of the outer square was 20 mm > *2d *in all models, since it was already shown [31] that for model size (boundaries of the outer square) being *2d *the error due to the finite size of the model is negligible.

Model geometries were meshed by triangular finite elements. The final mesh models were obtained refining the mesh until the discrepancies of the mean and maximum relative difference between numerical solutions, obtained with two different meshes were negligible. For example, for electrode configuration 2c the final mesh consisted of 86 944 elements. The results of this model were compared to the results obtained with the same electrode configuration but coarser mesh which consisted only of 21 736 elements. The relative difference of the mean and the maximum value of the electric field between the two models were 2.14 * 10^-6 ^and 4.22*10^-3^, respectively.

### Analytical calculations – plate electrodes

Analytical solution for the electric field between two infinite parallel plate electrodes (Fig. 1a) gives a trivial solution *E = U/d*, where *d *is the distance between the electrodes and *U *is the applied voltage on the electrodes. The electric field strength *E *is constant in the entire region between infinite electrodes.

### Analytical calculations – needle electrodes

As already shown [31], for electrostatic problem analytical solution for the potential and the electric field also around the needle electrodes in 2D can be obtained by solving Laplace equation, if the needle penetration depth is larger than the distance between the electrodes. If we consider Laplace equation of a complex analytic function for a given region:

Δ*φ*(*z*) = 0,     (1)

where *z *= *x *+ *iy*, we obtain that the real part of this function Re (*Φ*(*z*)) is also a solution of the Laplace equation. The potential can be written as a sum of multipoles of all electrodes, details are given in reference [31]. If higher terms in multipole series are neglected we can write the potential as a sum of the leading terms of all *n *electrodes:

φ(z)=∑n=1NCnlog⁡az−zn+C0,     (2)
 MathType@MTEF@5@5@+=feaafiart1ev1aaatCvAUfKttLearuWrP9MDH5MBPbIqV92AaeXatLxBI9gBaebbnrfifHhDYfgasaacH8akY=wiFfYdH8Gipec8Eeeu0xXdbba9frFj0=OqFfea0dXdd9vqai=hGuQ8kuc9pgc9s8qqaq=dirpe0xb9q8qiLsFr0=vr0=vr0dc8meaabaqaciaacaGaaeqabaqabeGadaaakeaaiiGacqWFgpGzcqGGOaakcqWG6bGEcqGGPaqkcqGH9aqpdaaeWbqaaiabdoeadnaaBaaaleaacqWGUbGBaeqaaOGagiiBaWMaei4Ba8Maei4zaC2aaSaaaeaacqWGHbqyaeaacqWG6bGEcqGHsislcqWG6bGEdaWgaaWcbaGaemOBa4gabeaaaaaabaGaemOBa4Maeyypa0JaeGymaedabaGaemOta4eaniabggHiLdGccqGHRaWkcqWGdbWqdaWgaaWcbaGaeGimaadabeaakiabcYcaSaaa@4AF2@

where *a *is the radius of an electrode, *z*_*n *_is the position of the n-th electrode and the coefficients *C*_*n *_are determined from the boundary conditions. The above approximation can be used when *a << d *(needle electrodes are not too thick compared to typical inter-electrode distance). From Eq. 2 we can obtain the electric field strength from calculating the gradient of the potential:

E(z)=∑n=1NCn1z−zn.     (3)
 MathType@MTEF@5@5@+=feaafiart1ev1aaatCvAUfKttLearuWrP9MDH5MBPbIqV92AaeXatLxBI9gBaebbnrfifHhDYfgasaacH8akY=wiFfYdH8Gipec8Eeeu0xXdbba9frFj0=OqFfea0dXdd9vqai=hGuQ8kuc9pgc9s8qqaq=dirpe0xb9q8qiLsFr0=vr0=vr0dc8meaabaqaciaacaGaaeqabaqabeGadaaakeaacqWGfbqrcqGGOaakcqWG6bGEcqGGPaqkcqGH9aqpdaaeWbqaaiabdoeadnaaBaaaleaacqWGUbGBaeqaaOWaaSaaaeaacqaIXaqmaeaacqWG6bGEcqGHsislcqWG6bGEdaWgaaWcbaGaemOBa4gabeaaaaaabaGaemOBa4Maeyypa0JaeGymaedabaGaemOta4eaniabggHiLdGccqGGUaGlaaa@42BB@

## Results

The results of our study are organized in five subsections. The first and second subsections show the numerical and analytical results of the electric field distribution, respectively, for plate and needle electrodes as shown in Figs. 1 and 2. In the third subsection we present the comparison of the numerical and analytical results. In the next subsection we quantify the local electric field for given electrode configurations. Finally, in the last subsection we analyze the effect of tissue inhomogeneities on the local electric field distribution for the needle electrode configurations.

**Figure 3:**
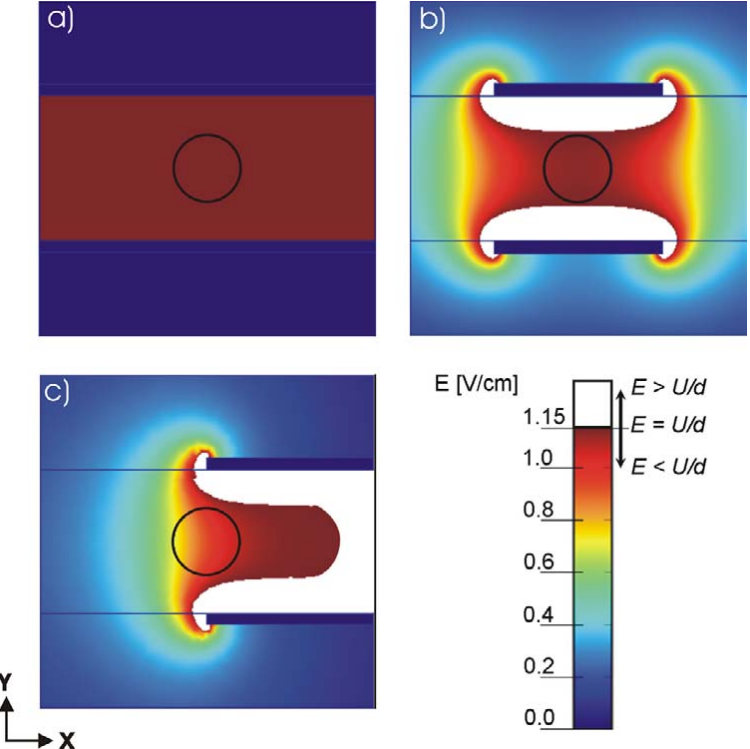
**Calculated electric field distribution for the geometries with parallel plate electrodes**. Numerical results of the electric field distribution for geometries defined in Fig. 1: a) the infinite plate electrodes case, b) the target tissue symmetrically placed between the finite plate electrodes and c) the non-symmetrical example when the target tissue is not entirely in-between the finite plate electrodes. The circle represents the target tissue e.g. tumor tissue and the white region represents part of tissue where *E *≥ *U/d*.

**Figure 4:**
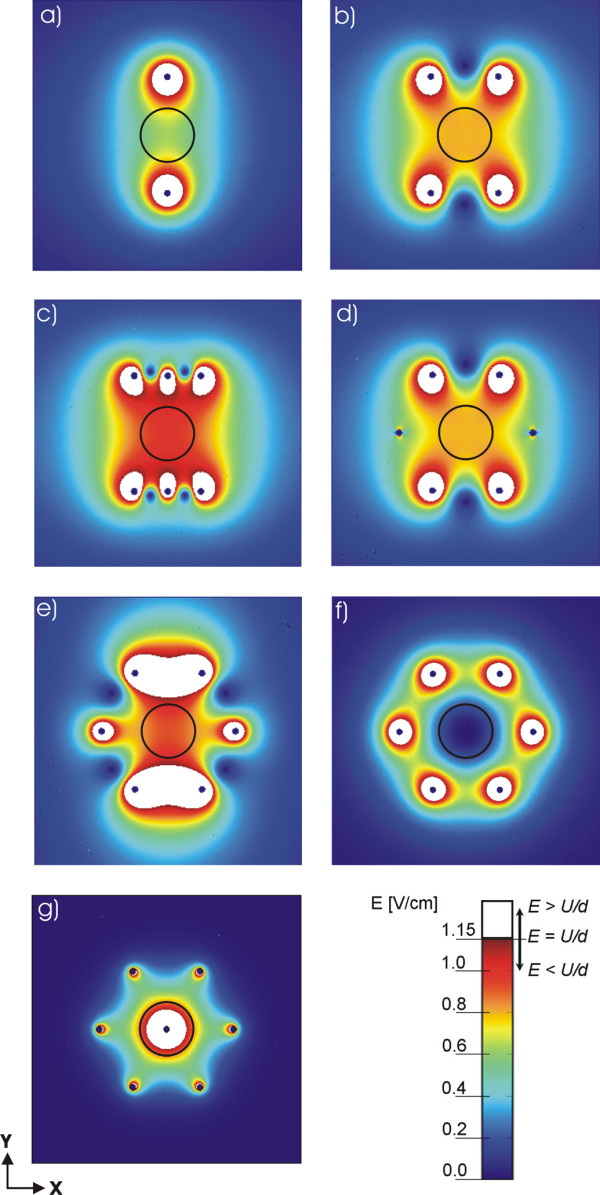
**Calculated electric field distribution for different needle electrode configurations**. Numerical results of the electric field distribution for the geometries defined in Fig. 2: a) two needle electrodes, b) four needle electrodes, c) six needle electrodes in two rows, d) six electrodes placed in a circle with polarities as shown in Fig. 2d, e) six needle electrodes placed in a circle with polarities as shown in Fig. 2e, f) seven needle electrodes placed in a circle – using alternating polarities seven needle electrodes placed in a circle – with central positive and surrounding electrodes having negative polarities and g) seven needle electrodes placed in a circle – with central positive and surrounding electrodes having negative polarities. In all cases the applied voltage was set in such a way that *U/d *= 1.15 V/cm, where d = 8.66 mm for Figs. 4a, 4b, 4c, 4d and 4e and d = 5 mm for Figs. 4f and 4g. The circle represents the target tissue and the white region represents part of tissue where *E *≥ *U/d*.

In order to compare and quantify the influence of geometry, number and position of different electrode configurations on the electric field distribution we used the same parameter *U*/*d *= 1.15 V/cm in all models. We present the calculated electric field with equal scale of *E *from 0 to 1.15 V/cm. The values of electric field strength are shown by colour scale legend (see Figs. 3 and 4) with the maximal value representing the ratio *U/d *= 1.15 V/cm in order to demonstrate the region below (color scale) and above the value *U/d *(white region). The encircled region in Figs. 3a–3c and Figs. 4a–4e represent one of the possible geometries and positions of the target tissues. It is within this target tissue that the electric field needs to be sufficiently high (*E > E*_*rev*_).

### Electric field distribution between plate and needle electrodes – numerical results

All models were calculated for the voltage between two electrodes *U *= (1 V, 0.575 V) giving the value of parameter *U*/*d *= 1.15 V/cm, but values of *E' *for any other voltage can be obtained just by multiplying all values with given voltage *U' *divided by applied voltage *U *(1 V, 0.575 V). Namely, since our models are linear all results for *E *can be scaled for any arbitrary applied voltage *U'*: *E' = E U'*/*U*. In the following subsections we will present results obtained for *U*/*d *= 1.15 V/cm and scaled results for *U/d *= 1300 V/cm.

#### I.) Plate electrodes

Fig. 3 presents the comparison of electric field distribution of three different configurations of parallel plate electrodes for *U *= 1 V and *d *= 8.66 mm (*U/d *= 1.15 V/cm). For an ideal case with infinite parallel plate electrodes (Fig. 3a) we obtained constant electric field in the entire region between the electrodes. In Fig. 3b we can see that for a more realistic geometry, where finite electrodes are considered, the electric field between the electrodes is not constant and is decreased towards the central region. Furthermore, if we change the position of electrodes with respect to the target tissue (encircled region), as presented in Fig. 3c, the electric field inside the target tissue is further reduced. Only in the case of the infinite parallel plate electrodes, one can use the expression *E = U/d*, and only in this ideal case *E *is constant in the entire region between the electrodes (provided that the tissue between the electrodes is homogeneous).

#### II.) Needle electrodes

In Fig. 4 numerically calculated electric field distribution for different needle electrode configurations and different polarities are shown. The values of the distances between the needle electrodes *d *and *l *are shown in Table 1, and were chosen in a way to correspond to values of Dev et al. [31]. The applied voltage for all configurations was *U *= 1 V (*d *= 8.66 mm), except for configurations shown in Fig. 2g and Fig. 2f where *U *= 0.575 V (*d *= 5 mm) keeping the ratio *U/d *constant.

**Table 1:** The distances *d *and *l *between the needle electrodes as defined in Fig. 2.

**Electrodes configuration**	**2** **Fig. 2a**	**4** **Fig. 2b**	**6** **Fig. 2c**	**6** **Fig. 2d, e**	**7** **Fig. 2f, g**
*d *[mm]	8.66	8.66	8.66	8.66	5
*l *[mm]	/	5	2.5	5	5

In Fig. 4 it can be clearly seen that the electric field distribution in the tissue strongly depends on the number, position and polarities of the electrodes. As expected the highest values of *E *are obtained in the vicinity of the electrodes. With increasing the number of electrodes the electric field strength inside the target tissue becomes higher. It can be seen that by using six or seven electrodes we can achieve a better distribution of *E *than by using only two or four electrodes. One can also observe that only a smaller part of the tissue is exposed to *E *≥ *U/d *(white region), whereas in the other regions of tissue *E *is smaller then *U/d*.

In Figs. 4d and 4e we compare the distribution of *E *for two different sets of polarities for electrode configuration of six electrodes arranged in the circle as used by Gilbert and co-workers [26]. We obtained higher electric field inside the target tissue with electrode configuration shown in Fig. 4e (3 positive, 3 negative electrodes) compared to the electric field inside the target tissue with the electrode configuration shown in Fig. 4d (2 positive, 2 negative electrodes). For both configurations the specific electrodes' positions enables rotation of the electric field direction (by rotating the polarities of the electrodes for a given angle) thus achieving better coverage of the target tissue with needed electric field. Comparing Figs. 4d to 4b we can also see that both electrode configuration results in equal electric field distribution, since the two electrodes with zero potential do not contribute to *E *distribution.

Figures 4f and 4g represent two examples of seven electrodes arranged in a circle with a central electrode having different polarities. We can see that in the first case (Fig. 4f) we obtain high intensity of the electric field in the ring around the electrodes surrounding the central region, whereas in the second case (Fig. 4g) we obtain high intensity of the electric field in the central region between the electrodes. Therefore by using combinations of these two different possibilities of setting the polarities of the electrodes we can successfully electropermeabilize all the tissue between the electrodes. However, by using only the configuration as shown in Fig. 4d the target tissue is not permeabilized.

### Electric field distribution between plate and needle electrodes – analytical results

#### I.) Plate electrodes

Analytical solution for the electric field between two infinite parallel plate electrodes (Fig. 1a) gives a trivial solution *E = U/d*, where *E *is constant in entire region. In all other cases *E *between the electrodes is not constant: for finite dimensions of the electrodes (Fig. 1b) or if the target tissue is not set entirely between the plate electrodes as shown in Fig. 1c.

#### II.) Needle electrodes

Since the derivation using the leading-order solution for a problem with electrodes positioned as shown in Fig. 2b is already given in detail in [31] we present here the final solutions for different geometries as shown in Fig. 2. In all geometries we set the applied voltage *U *by setting the potential on the electrodes to *V*_0 _= ± *U/2*. Using the equation for the potential Eq.2 (leading order approximation)

φ(r⇀)=∑n=1NCnlog⁡a|r⇀−r⇀n|+C0,     (4)
 MathType@MTEF@5@5@+=feaafiart1ev1aaatCvAUfKttLearuWrP9MDH5MBPbIqV92AaeXatLxBI9gBaebbnrfifHhDYfgasaacH8akY=wiFfYdH8Gipec8Eeeu0xXdbba9frFj0=OqFfea0dXdd9vqai=hGuQ8kuc9pgc9s8qqaq=dirpe0xb9q8qiLsFr0=vr0=vr0dc8meaabaqaciaacaGaaeqabaqabeGadaaakeaaiiGacqWFgpGzcqGGOaakcuWGYbGCgaGdaiabcMcaPiabg2da9maaqahabaGaem4qam0aaSbaaSqaaiabd6gaUbqabaGccyGGSbaBcqGGVbWBcqGGNbWzdaWcaaqaaiabdggaHbqaaiabcYha8jqbdkhaYzaaoaGaeyOeI0IafmOCaiNba4aadaWgaaWcbaGaemOBa4gabeaakiabcYha8baaaSqaaiabd6gaUjabg2da9iabigdaXaqaaiabd6eaobqdcqGHris5aOGaey4kaSIaem4qam0aaSbaaSqaaiabicdaWaqabaGccqGGSaalaaa@4E16@

and applying appropriate boundary condition (potential on the electrodes) we obtained the coefficients *C*_*n*_, which are given in Appendix section. Taking the real part of Eq. 3 and solutions for *C*_*n *_(Eqs. A.1-A.6) we obtained analytical expressions for the electric field strength for different geometries as shown in Fig. 2:

E(r⇀)=∑n=1NCn1|r⇀−rn⇀|.     (5)
 MathType@MTEF@5@5@+=feaafiart1ev1aaatCvAUfKttLearuWrP9MDH5MBPbIqV92AaeXatLxBI9gBaebbnrfifHhDYfgasaacH8akY=wiFfYdH8Gipec8Eeeu0xXdbba9frFj0=OqFfea0dXdd9vqai=hGuQ8kuc9pgc9s8qqaq=dirpe0xb9q8qiLsFr0=vr0=vr0dc8meaabaqaciaacaGaaeqabaqabeGadaaakeaacqWGfbqrcqGGOaakcuWGYbGCgaGdaiabcMcaPiabg2da9maaqahabaGaem4qam0aaSbaaSqaaiabd6gaUbqabaGcdaWcaaqaaiabigdaXaqaamaaemaabaWaa8HcaeaacqWGYbGCaiaawgniaiabgkHiTmaaFOaabaGaemOCai3aaSbaaSqaaiabd6gaUbqabaaakiaawgniaaGaay5bSlaawIa7aaaaaSqaaiabd6gaUjabg2da9iabigdaXaqaaiabd6eaobqdcqGHris5aOGaeiOla4caaa@4943@

The presented analytical results are extensions of the analytical expression given by Dev et al. [31] for geometries given in Fig. 2 for arbitrary values of *d *and *l*, as well as for different polarities in case of seven electrodes, where r⇀n
 MathType@MTEF@5@5@+=feaafiart1ev1aaatCvAUfKttLearuWrP9MDH5MBPbIqV92AaeXatLxBI9gBaebbnrfifHhDYfgasaacH8akY=wiFfYdH8Gipec8Eeeu0xXdbba9frFj0=OqFfea0dXdd9vqai=hGuQ8kuc9pgc9s8qqaq=dirpe0xb9q8qiLsFr0=vr0=vr0dc8meaabaqaciaacaGaaeqabaqabeGadaaakeaadaWhkaqaaiabdkhaYbGaayz0GaWaaSbaaSqaaiabd6gaUbqabaaaaa@3160@ is the position of the *n*-th electrode as shown Fig. 2. Using the analytical expression for the electric field (Eq.5 and Eqs. A.1-A.6) we calculated electric field distribution *E *for different electrode configurations. These analytical results arevery similar to numerical results of electric field distribution shown in Fig. 4.

### Comparison of the analytical and the numerical results

**Figure 5:**
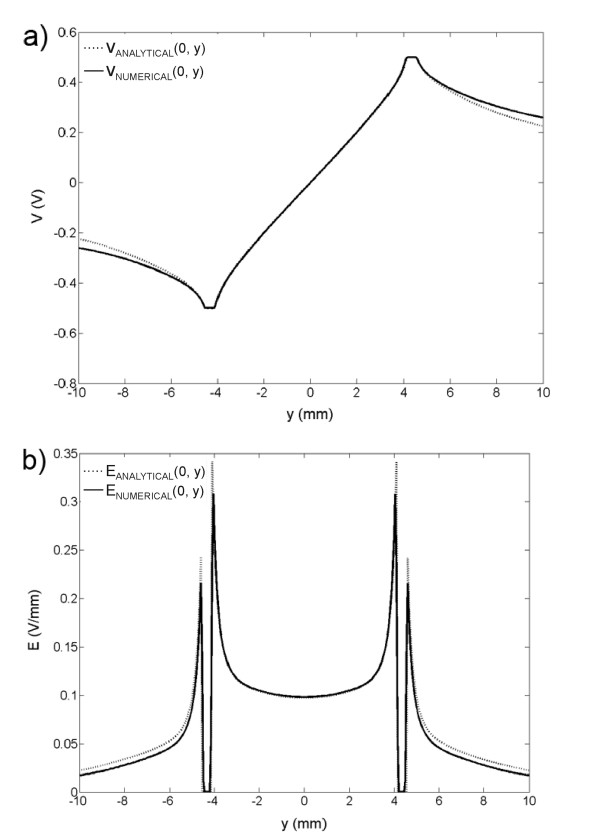
**Comparison of the analytical and the numerical solution**. The analytical and the numerical solutions of a) the electric potential and b) electric field distribution along *y *axis (*x *= *0*) for applied voltage *U *= 1 V (*V*_+ _= 0.5 V, *V*_- _= - 0.5 V) are given for the configuration defined in Fig. 2c.

In our study both numerical results as well as analytical results were obtained. The analytical results were validated with the numerical calculations for given electrode configurations. In Fig. 5 we compare the analytical and numerical solutions for the geometry of six electrodes (Fig. 2c) of the electric potential *V*(*x,y*) (a) and the electric field distribution *E*(*x,y*) (b) along *y *axis (*x *= *0*). We can see that a good agreement between numerical and analytical solution is obtained in the area between the electrodes, whereas the discrepancy between numerical and analytical solution increases outside the electrodes |y| > 4. The mean and maximal relative difference between numerical and analytical solutions of electric field strength inside the electrode array calculated between the electrodes (over all nodes within the area: |*x*| < 4 and |*y*| < 4) were less than 0.7 % and 3.9 %, respectively. Similarly, we obtained a good agreement for both, the potential and the electric field also for other presented geometries (results are not shown).

Since the differences between analytical and numerical results were negligible only numerical results are further analyzed and presented in figures.

### Quantification of the local electric field

In order to further quantify and compare local electric field distribution within the tissue for different electrodes and electrode configurations, as defined in Fig. 1 and Fig. 2, we calculated minimal *Ett*_*min *_and maximal electric field strengths *Ett*_*max *_inside the target tissue, as well as the highest value of *E *within entire tissue – *E*_*max*_. These parameters are important for the optimization of electrochemotherapy and gene electrotransfer, namely *Ett*_*min *_should be above *E*_*rev *_while *E*_*max *_should be as low as possible to prevent excessive damages of the surrounding tissue. Furthermore, we calculated the necessary voltage *U*_*c *_which has to be applied to the electrodes in order to achieve successful electropermeabilization in the target tissue *Ett*_*min *_≥ *E*_*rev*_, where we used *E*_*rev *_= *U/d*. Here we have to stress that we set the value *E*_*rev *_= *U/d *in order to compare our results of the local electric field distribution to the previously published studies which used the approximation *U/d *as an estimate of the local electric field in the treated tissue. The results of quantification of the parameters *Ett*_*min*_, *Ett*_*max *_and *E*_*max *_for given electrode configurations are listed in Tables 2, 3, 4, 5.

**Table 2:** Quantification of electric field strength for plate electrodes models – calculated *Ett*_*min*_, *Ett*_*max *_and *E*_*max *_parameters.

**2 plate electrode configuration**	**U/d = 1.15 V/cm**			**U/d = 1300 V/cm**		
	
	Target tissue Ett_max_(V/cm)	Target tissue Ett_min_(V/cm)	Entire tissue E_max_(V/cm)	Target tissue Ett_max_(V/cm)	Target tissue Ett_min_(V/cm)	Entire tissue E_max_(V/cm)
(Fig. 3a)	U/d = 1.15	U/d = 1.15	U/d = 1.15	U/d = 1300	U/d = 1300	U/d = 1300
(Fig. 3b)	1.152	1.113	5.515	1297.0	1253.0	6209.0
(Fig. 3c)	1.081	0.691	5.533	1217.0	777.9	6229.2

Calculated minimal *E *(*Ett*_*min*_) and maximal *E *(*Ett*_*max*_) inside the target tissue and maximal *E *within the entire tissue *E*_*max *_for different plate electrode configurations as defined in Fig. 1 are given. The results are given for applied voltage *U *= 1 V giving *U/d *= 1.15 V/cm and for applied voltage (scaled results) for *U *= 1125.83 V giving *U/d *= 1300 V/cm.

**Table 3:** Calculated values of *U*_*c *_and corresponding *Ett*_*min*_, *Ett*_*max *_and *E*_*max *_for plate electrodes.

**2 plate electrode configuration**	Target tissue Ett_max_(V/cm)	Target tissue Ett_min_(V/cm)	Entire tissue E_max_(V/cm)	Needed voltage on the electrodes-U_c _(V)
(Fig. 3a)	U/d = 1300	U/d = 1300	U/d = 1300	U = 1125.83
(Fig. 3b)	1345.8	U/d = 1300	6441.5	1168
(Fig. 3c)	2001	U/d = 1300	10242	1851

The needed voltage between the electrodes (*U*_*c*_) was chosen in a way that the minimal electric field inside the target tissue exceeded *U/d*: *Ett*_*min *_≥ *U/d*, thus assuring successful permeabilization of the entire target tissue.

**Table 4:** Quantification of electric field strength for needle electrode models – calculated *Ett*_*min*_, *Ett*_*max *_and *E*_*max *_parameters.

**Needle electrode configuration**	**U/d = 1.15 V/cm**			**U/d = 1300 V/cm**		
	
	Target tissue Ett_max_(V/cm)	Target tissue Ett_min_(V/cm)	Entire tissue E_max_(V/cm)	Target tissue Ett_max_(V/cm)	Target tissue Ett_min_(V/cm)	Entire tissue E_max_(V/cm)
2 (Fig. 4a)	0.804	0.527	6.618	905.4	591.7	7450.3
4 (Fig. 4b)	0.824	0.779	5.829	928.7	876.9	6562.5
6 (Fig. 4c)	1.049	0.911	5.166	1180.9	1025.2	5816.6
6 (Fig. 4d)	0.822	0.778	5.794	925.4	875.9	6523.1
6 (Fig. 4e)	1.064	0.835	7.443	1197.9	940.1	8379.6
7 (Fig. 4f)	0.21	~0	5.17	236.4	1.038	5820.8
7 (Fig. 4g)	8.1	0.84	8.1	9119.2	945.7	9119.2

The results for models shown in Figs. 4a – 4e were calculated for applied voltage *U *= 1 V (d=53 mm
 MathType@MTEF@5@5@+=feaafiart1ev1aaatCvAUfKttLearuWrP9MDH5MBPbIqV92AaeXatLxBI9gBaebbnrfifHhDYfgasaacH8akY=wiFfYdH8Gipec8Eeeu0xXdbba9frFj0=OqFfea0dXdd9vqai=hGuQ8kuc9pgc9s8qqaq=dirpe0xb9q8qiLsFr0=vr0=vr0dc8meaabaqaciaacaGaaeqabaqabeGadaaakeaacqWGKbazcqGH9aqpcqaI1aqndaGcaaqaaiabiodaZaWcbeaakiabbccaGiabb2gaTjabb2gaTbaa@349D@) and for models shown in Figs. 4f – 4g for applied voltage *U *= 0.575 V (*d *= *l *= 5 mm) giving *U/d *= 1.15 V/cm. Furthermore, by scaling the results we also calculated the parameters *Ett*_*min*_, *Ett*_*max *_and *E*_*max *_for *U/d *= 1300 V/cm.

**Table 5:** Calculated values of *U*_*c *_and corresponding *Ett*_*min*_, *Ett*_*max *_and *E*_*max *_for needle electrodes.

**Needle electrode Configuration**	Target tissue Ett_max_(V/cm)	Target tissue Ett_min_(V/cm)	Entire tissue E_max_(V/cm)	Needed voltage on the electrodes-U_c _(V)
2 (Fig. 4a)	1983.3	U/d = 1300	16325.0	2466.8
4 (Fig. 4b)	1376.8	U/d = 1300	9727.4	1668.8
6 (Fig. 4c)	1496.9	U/d = 1300	7371.9	1427.0
6 (Fig. 4d)	1373.2	U/d = 1300	9625	1670.0
6 (Fig. 4f)	1653.7	U/d = 1300	11558.7	1557.0
7 (Fig. 4f) *	/	/	/	/
7 (Fig. 4g)	12536	U/d = 1300	12536	889.88

*With this specific configuration of polarities (Fig. 4f) we can not achieve *Et*_*min*_* = U/d (Et*_*min*_*~0)*, since the electric field intensity inside the target tissue is almost zero (*Et*_*min*_~0) and therefore is this configuration suitable only in a combination with the configuration as shown in Fig. 4g.

#### I.) Plate electrodes

From Fig. 1 and Table 2 it can be seen that electric field is homogeneous (*Ett*_*max*_*= Ett*_*min*_*= E*_*max*_*= U/d) *only for the model with infinite plate electrodes and can be calculated as the ratio *E = U/d*. As soon as we use more realistic electrodes having finite length *l *(see Fig. 1b), or realistic electrodes position with the respect to the treated tissue (see Fig. 1c), the electric field intensity within the tissue between the electrodes is no longer homogeneous. The values of *Ett*_*min *_and *Ett*_*max *_inside the target tissue have lower values from *U/d *whereas the *E*_*max *_in the near proximity of the plate electrodes increases and are higher than ratio *U/d *(see Table 2). In Table 3 we give the results of necessary voltage *U*_*c *_in order to obtain the condition *Ett*_*min *_> *U/d*, needed for successful target tissue permeabilization. Based on this we can conclude that in realistic cases (see Figs. 1b and 1c) the value of *U*_*c *_has to be higher compared to the value *U*_*c *_in the homogeneous model (Fig. 1a) in order to effectively treat the entire target tissue.

#### II.) Needle electrodes

In Fig. 4 we compare electric field distributions calculated numerically using FEM method for different needle electrode configurations and polarities. In order to obtain the parameter *U/d *= 1.15 V/cm in all models we set the applied voltage U=1 V(U/d=1V/53 mm
 MathType@MTEF@5@5@+=feaafiart1ev1aaatCvAUfKttLearuWrP9MDH5MBPbIqV92AaeXatLxBI9gBaebbnrfifHhDYfgasaacH8akY=wiFfYdH8Gipec8Eeeu0xXdbba9frFj0=OqFfea0dXdd9vqai=hGuQ8kuc9pgc9s8qqaq=dirpe0xb9q8qiLsFr0=vr0=vr0dc8meaabaqaciaacaGaaeqabaqabeGadaaakeaacqWGvbqvcqGH9aqpcqaIXaqmcqqGGaaicqqGwbGvcqGGOaakcqWGvbqvcqGGVaWlcqWGKbazcqGH9aqpcqaIXaqmcqqGwbGvcqGGVaWlcqaI1aqndaGcaaqaaiabiodaZaWcbeaakiabbccaGiabb2gaTjabb2gaTbaa@3FBA@ for electrode configurations shown in Figs. 4a–4e and for models shown in Figs. 4f–4g for applied voltage *U *= 0.575 V (*d *= *l *= 5 mm) giving *U/d *= 1.15 V/cm.

As shown in Figs. 4d–4g, we obtained that by using several electrodes (six or seven electrodes in the circle) and changing the potential and polarity on the electrodes we can achieve better coverage of target tissue with adequate *E*. In the case of only two electrodes we can see that the *E *in the surrounding tissue can be too high and may cause irreversible damages (Fig. 4a). In the cases of two, four and six electrodes (Figs. 4a–4c) reversing the polarities does not change the electric field distribution. Nevertheless, reversing the polarity can improve electropermeabilization on the level of cell membrane since the orientation of the electric field determines which side of the cell will be more permeabilized [23,31,42-44].

In table 4 we compare different configurations of the needle electrodes. If we compare these values to the "electric field intensity" *U/d*, we can see that both maximal and minimal *E *deviate significantly from *U/d *value, which can be seen also in Fig 4. The low values of *Ett*_*min *_mean that some parts of the target tissue will not be permeabilized whereas some parts of the 0surrounding tissue might be exposed to too high values causing irreversible damage especially around the electrodes (too high *E*_*max*_), which is most pronounced for the geometry with two needle electrodes. From Table 4 it can be seen that for four and six electrodes *Ett*_*min *_increases while *E*_*max *_decreases. We also calculated the needed voltage *U*_*c *_(Table 5) which has to be applied on the electrodes in order to achieve the condition *Ett*_*min *_≥ *U/d *assuming target tissue permeabilization and as it can be seen from Table 5 the needed voltage *U*_*c *_differs substantially for different needle electrode configurations. Namely, increasing the number of electrodes from two to six we can decrease the applied voltage *U*_*c *_from 2467 V to 1427 V.

### The effect of tissue inhomogeneities on the electric field distribution

**Figure 6:**
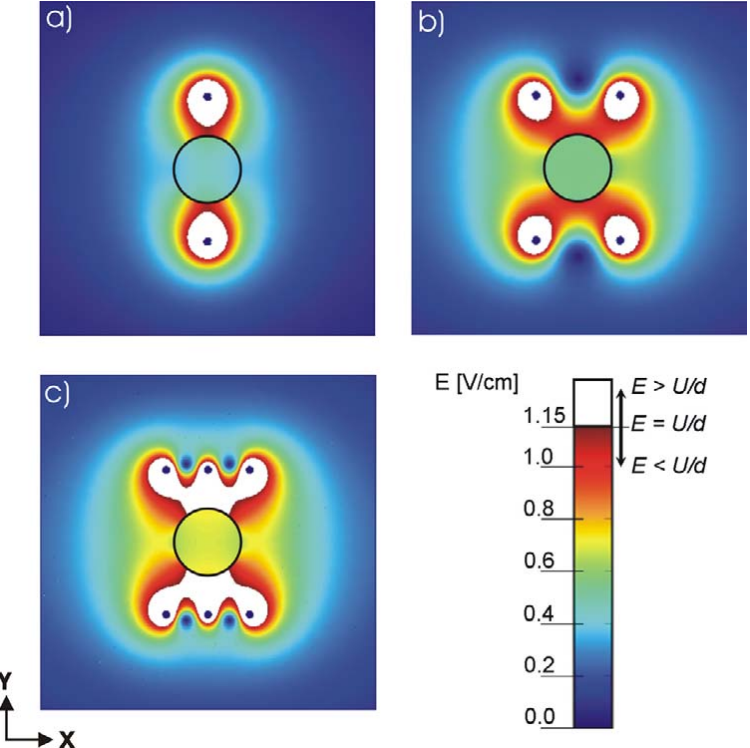
**Calculated electric field distribution for in-homogeneous models**. Numerical results of the electric field distribution for needle electrode configurations defined in Figs. 2a-2c: a) two needle electrodes, b) four needle electrodes, c) six needle electrodes in two rows taking into account two-times higher conductivity of the target tissue compared to surrounding tissue (conductivity of the target tissue is *σtt *= 0.4 S/m and conductivity of the surrounding tissue *σst *= 0.2 S/m). In all cases the applied voltage was set in such a way that *U/d *= 1.15 V/cm.

In order to analyze possible effects of tissue inhomogeneities we made additional models where target tissue had increased conductivity which is based on the fact that the tumor tissue has in general higher conductivity than its surrounding tissue. Namely, from the literature [19,45] we determined that reasonable approximation for conductivity of the target (tumor) tissue is *σtt *= 0.4 S/m and conductivity of the surrounding tissue *σst *= 0.2 S/m. In Fig. 6 we compare electric field distributions calculated numerically using FEM method for two, four needle electrodes and six needle electrodes taking into account higher conductivity of the target tissue compared to the surrounding tissue.

Comparing the results of the inhomogeneous models shown in Fig. 6 to the electric field distribution in homogeneous models (Figs. 4a–4c) we obtained that in the inhomogeneous model the electric field strength inside the target tissue is lower, while the larger portion of the surrounding tissue is exposed to the value exceeding *U/d*. However, similarly as in homogenous model we again obtained that with larger number of electrodes better coverage of the target tissue with adequate *E *is obtained, namely for larger number of electrodes *Ett*_*min *_increases while *E*_*max *_decreases (see Table 6). We also obtained that similarly as for homogeneous models the local electric field is significantly different from the value *U/d*, e.g. for the selected parameters the minimum electric field strength inside the target tissue can deviate from the *U/d *by more than factor 3, see the Table 6.

**Table 6:** Quantification of the electric field strength for in-homogeneousmodels – calculated *Ett*_*min*_, *Ett*_*max *_and *E*_*max *_parameters.

**Needle electrode configuration**	**U/d = 1.15 V/cm**			**U/d = 1300 V/cm**		
	
	Target tissue Ett_max_(V/cm)	Target tissue Ett_min_(V/cm)	Entire tissue E_max_(V/cm)	Target tissue Ett_max_(V/cm)	Target tissue Ett_min_(V/cm)	Entire tissue E_max_(V/cm)
2 (Fig. 6a)	0.558	0.364	7.001	628.05	409.9	7887.1
4 (Fig. 6b)	0.572	0.539	6.104	643.4	606.2	6872.5
6 (Fig. 6c)	0.741	0.639	5.339	833.9	718.9	6011.2

Quantification of the electric field strength (*E*) for in-homogeneous models with needle electrode configurations which are defined in Fig. 2a-c. Conductivity of the target tissue is *σtt *= 0.4 S/m and the conductivity of the surrounding tissue *σst *= 0.2 S/m. Calculated minimal *E *(*Ett*_*min*_) and maximal *E *(*Ett*_*max*_) inside the target tissue and maximal *E *within the entire tissue *E*_*max *_for different needle electrode configurations. The results for models shown in Figs. 6a-6c were calculated for applied voltage *U *= 1 V (d=53 mm
 MathType@MTEF@5@5@+=feaafiart1ev1aaatCvAUfKttLearuWrP9MDH5MBPbIqV92AaeXatLxBI9gBaebbnrfifHhDYfgasaacH8akY=wiFfYdH8Gipec8Eeeu0xXdbba9frFj0=OqFfea0dXdd9vqai=hGuQ8kuc9pgc9s8qqaq=dirpe0xb9q8qiLsFr0=vr0=vr0dc8meaabaqaciaacaGaaeqabaqabeGadaaakeaacqWGKbazcqGH9aqpcqaI1aqndaGcaaqaaiabiodaZaWcbeaakiabbccaGiabb2gaTjabb2gaTbaa@349D@). Furthermore, by scaling the results we also calculated the parameters *Ett*_*min*_, *Ett*_*max *_and *E*_*max *_for *U/d *= 1300 V/cm.

In Table 7 we give the results of the necessary voltage *U*_*c *_in order to meet the condition *Ett*_*min*_*> U/d *for inhomogeneous models where *σtt *is higher then *σst*. Our results show that *U*_*c *_for given inhomogeneous model (*σtt *= 2 × *σst*) has to be higher compared to *U*_*c *_in the homogeneous models in order to effectively treat the entire target tissue.

**Table 7:** Calculated values of *U*_*c *_and corresponding *Ett*_*min*_, *Ett*_*max *_and *E*_*max *_for in-homogeneous models (Figs. 6a-6c).

**Needle electrode configuration**	Target tissue Ett_max_(V/cm)	Target tissue Ett_min_(V/cm)	Entire tissue E_max_(V/cm)	Needed voltage on the electrodes-U_c _(V)
2 (Fig. 6a)	1991.87	U/d = 1300	25014.0	3570.6
4 (Fig. 6b)	1379.80	U/d = 1300	14738.4	2414.4
6 (Fig. 6c)	1508.0	U/d = 1300	10870.4	2035.9

Specific conductivity of the target tissue is *σtt *= 0.4 S/m and specific conductivity of the surrounding tissue *σst *= 0.2 S/m.

## Discussion

In this study we numerically and analytically determined and compared the local electric field distribution in 2D for different electrode configurations which are used for in vivo electrochemotherapy and gene electrotransfer. We quantify and compare the local electric field by means of three parameters: the maximal in minimal local electric fields inside the treated tissue – *Ett*_*min*_, *Ett*_*max *_and maximal *E *over the entire treated tissue – *E*_*max*_. Namely, the criteria for adequate or »optimal« local *E *distribution are the following: i) all the target tissue has to be exposed to the *E *above the threshold value for reversible electroporation (*Ett*_*min*_*> E*_*rev*_); ii) the maximal *E *inside the target tissue *Ett*_*max *_has to be below the threshold value for irreversible electroporation (*Ett*_*max*_*< E*_*irrev*_), which is specially important in gene electrotransfer and iii) the surrounding tissue should not be exposed to excessively high electric field, therefore the maximal electric field in entire tissue *E*_*max *_should be as low as possible, while meeting the first condition *Ett*_*min *_>*E*_*rev*_.

We further calculated the needed voltage *U*_*c *_(Table 5) which has to be applied on the electrodes in order to subject the entire target tissue to the sufficiently high local electric field (*Ett*_*min *_≥ *U/d*), where the value *U/d *was used in order to compare this parameter to the actual magnitude of *E *inside the treated tissue.

We showed that the electric field distribution in the tissue strongly depends on the number and position of the electrodes, as well as of the electric field orientation, as demonstrated in Fig. 4. As expected the highest values of *E *are obtained in the vicinity of the electrodes where *E *can exceed the irreversible threshold value *E*_*irrev *_leading to the damage of the tissue. With increasing the number of electrodes the electric field strength inside the target tissue becomes higher for the same voltage applied, e.g. from the Table 4 it can be seen that *Ett*_*min *_increases and *E*_*max *_decreases for higher number of electrodes. Considering that *Ett*_*min *_should be above *E*_*rev*_, while keeping *E*_*max *_as low as possible it can be seen (Table 4) that the six electrode configurations have the best ratio between *Ett*_*min *_and *E*_*max*_. Configurations with seven electrodes are reasonable only when combining the two polarities settings (Figs. 4f and 4f) on the electrodes in order to electropermeabilize the larger area of treated tissue.

We also demonstrate that if parameter *U/d *is used to select the applied voltage only smaller part of the tissue is exposed to *E *≥ *U/d *(white region in Fig. 4), whereas in the other regions of tissue *E *is too small. We obtained that the ratio between minimal *E *inside the target tissue (*Ett*_*min*_) and the value *U/d *can deviate for more than a factor of 2 (see Table 4). The higher local electric field can be achieved by increasing the applied voltage, therefore we further calculate the needed voltage *U*_*c *_to fulfill the condition *Ett*_*min*_*> U/d *over the entire target tissue. We showed that the needed applied voltage *U*_*c *_differs substantially for different needle electrode configurations (Table 5). Thus, the electric field distribution strongly depends on geometry and position of electrodes with respect to the target tissue therefore the needed voltage (*U*_*c*_) requires its own calculation for each individual configuration. From Table 5 it can be seen that the *U*_*c *_for two needle electrodes has to be about 2400 V compared to other configurations where *U*_*c *_is in the range from 1400 V to 1700 V.

Another possibility to achieve better coverage of the target tissue with the adequate *E *with the same applied voltage is changing the electric field orientation as already experimentally and numerically demonstrated with two 90° rotations of *E *using plate electrodes in [23] and experimentally in [26] with a sequence of 60° rotations of *E *using needle electrode configurations, as shown in Figs. 2d and 2e.

Moreover, changing the electric field orientation during the electric pulse delivery is also important for gene electrotransfer as it improves the efficiency of gene electrotransfer indirectly by also increasing the membrane area available for the transfer of plasmid DNA [46].

We used 2D numerical and analytical models in order to compare *E *for different electrode configurations in the central plane of a more general 3D model. The presented 2D results are good approximation of local electric field distribution in 3D models for needle electrodes since electrodes are usually long and deeply inserted in tissue.

The presented analytical solutions in 2D for the electric field around needle electrodes are extensions of the analytical expressions given by Dev et al. [31] for geometries given in Fig. 2 for arbitrary values of *d *and *l*, as well as for different polarities in case of six and seven electrodes. By comparing numerical and analytical calculations for given needle electrode configurations we obtained good agreement between the two methods. Thus we showed that the leading-order analytical approximation accurately describes the electric field distribution in the region between the needle electrodes. The presented analytical solutions can be used as a rapid pre-analysis of the electric field distribution for different needle electrode configurations.

Our models are approximation of more complex and in general time-dependent models where one has to take into account also the increase of the effective conductivity of the permeabilized region [18,19,47-51]. In our present study we assumed that tissue has a constant value of conductivity which represents the final stage of electropermeabilization. In most of the models we assumed homogeneous properties of the treated tissue which neglects the differences of the conductivities for different tissues. For plate electrodes, which are usually placed on the skin, this approximation is not adequate since the conductivity of the skin is few orders of magnitude lower [19]. However, for needle electrodes homogeneous models can be used to compare different configurations, since the treated tissues have roughly similar conductivities [45] and we can use the average conductivity.

In order to analyze possible effects of tissue inhomogeneities we made additional numerical models where target tissue had increased conductivity. The main conclusions of our study are independent of the electrical properties of tissues either homogenous or inhomogeneous. We obtained that similarly as for homogeneous models electric field distribution significantly depends on the configuration and that the deviation of the value *U/d *approximation from local *E *inside the target tissue can be even more pronounced. Furthermore, also for inhomogeneous models six electrodes result in better local electric field distribution in terms of achieving high *Ett*_*min *_and relatively low *E*_*max *_compared to two or four needle electrodes models (Table 6).

## Conclusion

The main objective of this paper was to provide the solutions of local electric field distribution and to visualize the local electric field inside the target tissue for most commonly used electrode configurations in electrochemotherapy and gene electrotransfer. In presented study we numerically and analytically quantify and compare electric field distribution in 2D for different electrode configurations which are used for in vivo electrochemotherapy and gene electrotransfer for the same value of parameter *U/d*. We demonstrate that the calculated local electric field inside the target tissue strongly depends on the chosen electrodes and electrode configuration and can be significantly different from a gross approximation *U/d *as usually used as an estimate of the local electric field in a number of different reports [8-10,12,15,32-36].

We show that electric field distribution strongly depends on geometry, position and polarity of the electrodes with respect to target tissue and that it requires its own calculation for each individual configuration, which is in agreement with previous reports [12,15,17,25,26,32,34,40,41,52]. We present visualization of the electric field distribution and quantification of the maximal and minimal values of *E *inside the target tissue for frequently used electrode configurations. We also calculate the needed voltage for a specific configuration to meet the criterion that the local electric field over the entire target tissue exceeds the threshold value.

The results show that higher electric field inside the target tissue can be obtained by increasing the number of the electrodes, e.g. we obtained better electric field distribution with six electrodes compared to four or two electrodes (see Figs. 4a–4c). Namely, in this way the local electric field in the target tissue is increased while the electric field inside the surrounding tissue is reduced. We further show that changing the orientation of the electric field by changing electrodes' polarities leads to better coverage of the target tissue with desirable local electric field, which was already proven experimentally to improve electrochemotherapy efficiency and gene electrotransfer [23,34,43,46]. For example by consecutive changing the polarities of the electrodes (i.e. combining the polarity configurations Fig. 4f and Fig. 4g) we electropermeabilize larger area with the same electrode configurations.

In addition we showed that for needle electrode configuration we can use the analytical solution as a rapid and simple method for visualizing electric field distribution inside the tissue without using special software for numerical modeling. But in case of more complex geometries and inhomogeneities of the tissue, numerical modeling is required to determine optimal parameters in order to achieve efficient tissue permeabilization [15-17,25,49,50,52].

To conclude, our numerical models and analytical calculations provide an estimate of actual local *E *inside the target tissue and can be used for comparison of different electrode configurations. They also enable more precise choice of applied voltage compared to using *U/d *approximation. Since optimal geometry, arrangement and position of the electrodes strongly depend on the position and geometry of the target tissue it is of crucial importance to design a system of electrodes, which could be easily adjustable according to each individual case and to develop software for numerical calculation which would enable optimization of parameters in order to render electrochemotherapy and gene electrotransfer as efficient as possible. An important step towards the optimization of local electric field for effective ECT has been made recently by IGEA company [53] currently providing the electroporator designed specifically to be used in the clinical practice for electrochemotherapy. They provide the voltage for different distances between electrodes taking into account also the differences in local electric field distribution for different electrode configurations. In order to improve the efficiency of the treatments training sessions should be also involved. The training sessions should also provide educational material about the knowledge and experiences that have already been acquired with electrochemotherapy and gene electrotransfer. This can be brought about by the web technology, as an easy and important way to collect and organize the information obtained from different clinical and research centers [54-56].

## Appendix

In this section we present solutions of the Laplace equation for the coefficients *C*_*n *_from Eq. 4 for different needle electrode configurations. We obtained the following result for two needle electrodes (Fig. 2a):

C1=−C2=V0log⁡(d/a),     (A.1)
 MathType@MTEF@5@5@+=feaafiart1ev1aaatCvAUfKttLearuWrP9MDH5MBPbIqV92AaeXatLxBI9gBaebbnrfifHhDYfgasaacH8akY=wiFfYdH8Gipec8Eeeu0xXdbba9frFj0=OqFfea0dXdd9vqai=hGuQ8kuc9pgc9s8qqaq=dirpe0xb9q8qiLsFr0=vr0=vr0dc8meaabaqaciaacaGaaeqabaqabeGadaaakeaacqWGdbWqdaWgaaWcbaGaeGymaedabeaakiabg2da9iabgkHiTiabdoeadnaaBaaaleaacqaIYaGmaeqaaOGaeyypa0ZaaSaaaeaacqWGwbGvdaWgaaWcbaGaeGimaadabeaaaOqaaiGbcYgaSjabc+gaVjabcEgaNnaabmaabaGaemizaqMaei4la8IaemyyaegacaGLOaGaayzkaaaaaiabcYcaSaaa@4083@

for four electrodes (Fig. 2b):

C1=C2=−C3=−C4=V0log⁡(d2+l2lda),     (A.2)
 MathType@MTEF@5@5@+=feaafiart1ev1aaatCvAUfKttLearuWrP9MDH5MBPbIqV92AaeXatLxBI9gBaebbnrfifHhDYfgasaacH8akY=wiFfYdH8Gipec8Eeeu0xXdbba9frFj0=OqFfea0dXdd9vqai=hGuQ8kuc9pgc9s8qqaq=dirpe0xb9q8qiLsFr0=vr0=vr0dc8meaabaqaciaacaGaaeqabaqabeGadaaakeaacqWGdbWqdaWgaaWcbaGaeGymaedabeaakiabg2da9iabdoeadnaaBaaaleaacqaIYaGmaeqaaOGaeyypa0JaeyOeI0Iaem4qam0aaSbaaSqaaiabiodaZaqabaGccqGH9aqpcqGHsislcqWGdbWqdaWgaaWcbaGaeGinaqdabeaakiabg2da9maalaaabaGaemOvay1aaSbaaSqaaiabicdaWaqabaaakeaacyGGSbaBcqGGVbWBcqGGNbWzdaqadaqcaawaaOWaaSaaaKaaGfaakmaakaaajaaybaGaemizaqMcdaahaaqcbawabeaacqaIYaGmaaqcaaMaey4kaSIaemiBaWMcdaahaaqcbawabeaacqaIYaGmaaaabeaaaKaaGfaacqWGSbaBaaGcdaWcaaqcaawaaiabdsgaKbqaaiabdggaHbaaaiaawIcacaGLPaaaaaGccqGGSaalaaa@51DD@

for six electrodes (arranged in two parallel rows of three electrodes in each), as shown in Fig. 2c:

C1=C3=−C4=−C6=V0log⁡(d/a)−log⁡(d2+l2/l)log⁡(d/a)log⁡(dd2+4l2/2al)−2(log⁡(d2+l2/l))2,C2=−C5=V0−2C1log⁡(d2+l2/l)log⁡(d/a),     (A.3)
 MathType@MTEF@5@5@+=feaafiart1ev1aaatCvAUfKttLearuWrP9MDH5MBPbIqV92AaeXatLxBI9gBaebbnrfifHhDYfgasaacH8akY=wiFfYdH8Gipec8Eeeu0xXdbba9frFj0=OqFfea0dXdd9vqai=hGuQ8kuc9pgc9s8qqaq=dirpe0xb9q8qiLsFr0=vr0=vr0dc8meaabaqaciaacaGaaeqabaqabeGadaaakeaafaqabeGabaaabaGaem4qam0aaSbaaSqaaiabigdaXaqabaGccqGH9aqpcqWGdbWqdaWgaaWcbaGaeG4mamdabeaakiabg2da9iabgkHiTiabdoeadnaaBaaaleaacqaI0aanaeqaaOGaeyypa0JaeyOeI0Iaem4qam0aaSbaaSqaaiabiAda2aqabaGccqGH9aqpcqWGwbGvdaWgaaWcbaGaeGimaadabeaakmaalaaabaGagiiBaWMaei4Ba8Maei4zaCMaeiikaGIaemizaqMaei4la8IaemyyaeMaeiykaKIaeyOeI0IagiiBaWMaei4Ba8Maei4zaCMaeiikaGYaaOaaaeaacqWGKbazdaahaaWcbeqaaiabikdaYaaakiabgUcaRiabdYgaSnaaCaaaleqabaGaeGOmaidaaaqabaGccqGGVaWlcqWGSbaBcqGGPaqkaeaacyGGSbaBcqGGVbWBcqGGNbWzcqGGOaakcqWGKbazcqGGVaWlcqWGHbqycqGGPaqkcyGGSbaBcqGGVbWBcqGGNbWzcqGGOaakcqWGKbazdaGcaaqaaiabdsgaKnaaCaaaleqabaGaeGOmaidaaOGaey4kaSIaeGinaqJaemiBaW2aaWbaaSqabeaacqaIYaGmaaaabeaakiabc+caViabikdaYiabdggaHjabdYgaSjabcMcaPiabgkHiTiabikdaYmaabmaabaGagiiBaWMaei4Ba8Maei4zaCMaeiikaGYaaOaaaeaacqWGKbazdaahaaWcbeqaaiabikdaYaaakiabgUcaRiabdYgaSnaaCaaaleqabaGaeGOmaidaaaqabaGccqGGVaWlcqWGSbaBcqGGPaqkaiaawIcacaGLPaaadaahaaWcbeqaaiabikdaYaaaaaGccqGGSaalaeaacqWGdbWqdaWgaaWcbaGaeGOmaidabeaakiabg2da9iabgkHiTiabdoeadnaaBaaaleaacqaI1aqnaeqaaOGaeyypa0ZaaSaaaeaacqWGwbGvdaWgaaWcbaGaeGimaadabeaakiabgkHiTiabikdaYiabdoeadnaaBaaaleaacqaIXaqmaeqaaOGagiiBaWMaei4Ba8Maei4zaCMaeiikaGYaaOaaaeaacqWGKbazdaahaaWcbeqaaiabikdaYaaakiabgUcaRiabdYgaSnaaCaaaleqabaGaeGOmaidaaaqabaGccqGGVaWlcqWGSbaBcqGGPaqkaeaacyGGSbaBcqGGVbWBcqGGNbWzcqGGOaakcqWGKbazcqGGVaWlcqWGHbqycqGGPaqkaaGaeiilaWcaaaaa@ABD2@

for six electrodes arranged in circle (Fig. 2e):

C1=C5=−C2=−C4=V0[2log⁡(3)+log⁡(a/2d)],−C3=C6=2C1log⁡(3)−V0log⁡(a/2d),     (A.4)
 MathType@MTEF@5@5@+=feaafiart1ev1aaatCvAUfKttLearuWrP9MDH5MBPbIqV92AaeXatLxBI9gBaebbnrfifHhDYfgasaacH8akY=wiFfYdH8Gipec8Eeeu0xXdbba9frFj0=OqFfea0dXdd9vqai=hGuQ8kuc9pgc9s8qqaq=dirpe0xb9q8qiLsFr0=vr0=vr0dc8meaabaqaciaacaGaaeqabaqabeGadaaakeaafaqabeGabaaabaGaem4qam0aaSbaaSqaaiabigdaXaqabaGccqGH9aqpcqWGdbWqdaWgaaWcbaGaeGynaudabeaakiabg2da9iabgkHiTiabdoeadnaaBaaaleaacqaIYaGmaeqaaOGaeyypa0JaeyOeI0Iaem4qam0aaSbaaSqaaiabisda0aqabaGccqGH9aqpcqWGwbGvdaWgaaWcbaGaeGimaadabeaakmaadmaabaGaeGOmaiJagiiBaWMaei4Ba8Maei4zaCMaeiikaGYaaOaaaeaacqaIZaWmaSqabaGccqGGPaqkcqGHRaWkcyGGSbaBcqGGVbWBcqGGNbWzcqGGOaakcqWGHbqycqGGVaWlcqaIYaGmcqWGKbazcqGGPaqkaiaawUfacaGLDbaacqGGSaalaeaacqGHsislcqWGdbWqdaWgaaWcbaGaeG4mamdabeaakiabg2da9iabdoeadnaaBaaaleaacqaI2aGnaeqaaOGaeyypa0ZaaSaaaeaacqaIYaGmcqWGdbWqdaWgaaWcbaGaeGymaedabeaakiGbcYgaSjabc+gaVjabcEgaNjabcIcaOmaakaaabaGaeG4mamdaleqaaOGaeiykaKIaeyOeI0IaemOvay1aaSbaaSqaaiabicdaWaqabaaakeaacyGGSbaBcqGGVbWBcqGGNbWzcqGGOaakcqWGHbqycqGGVaWlcqaIYaGmcqWGKbazcqGGPaqkaaGaeiilaWcaaaaa@73B6@

for seven electrodes, as shown in Fig. 2f (six electrodes arranged in circle with additional placed in the center of this circle):

C1,3,5=−C2,4,6=V0log⁡(2d/3a),C7=0.     (A.5)
 MathType@MTEF@5@5@+=feaafiart1ev1aaatCvAUfKttLearuWrP9MDH5MBPbIqV92AaeXatLxBI9gBaebbnrfifHhDYfgasaacH8akY=wiFfYdH8Gipec8Eeeu0xXdbba9frFj0=OqFfea0dXdd9vqai=hGuQ8kuc9pgc9s8qqaq=dirpe0xb9q8qiLsFr0=vr0=vr0dc8meaabaqaciaacaGaaeqabaqabeGadaaakeaafaqabeqacaaabaGaem4qam0aaSbaaSqaaiabigdaXiabcYcaSiabiodaZiabcYcaSiabiwda1aqabaGccqGH9aqpcqGHsislcqWGdbWqdaWgaaWcbaGaeGOmaiJaeiilaWIaeGinaqJaeiilaWIaeGOnaydabeaakiabg2da9maalaaabaGaemOvay1aaSbaaSqaaiabicdaWaqabaaakeaacyGGSbaBcqGGVbWBcqGGNbWzdaqadaqaaiabikdaYiabdsgaKjabc+caViabiodaZiabdggaHbGaayjkaiaawMcaaaaacqGGSaalaeaacqWGdbWqdaWgaaWcbaGaeG4naCdabeaakiabg2da9iabicdaWiabc6caUaaaaaa@4EEB@

In all configurations 2a–2f is the number of positive and negative electrodes equal, so we can set *C*_0 _= 0.

For seven electrodes as shown in Fig. 2g we have one positive and six negative electrodes, so *C*_0 _is not zero. To satisfy conservation of the current we have additional condition *C*_7 _= -6*C*_1..6_, and thus we obtain:

C1..6=−2V06log⁡(a5/6d5)−12log⁡(a/d),C7=−6C1..6,C0=V0−6C1..6log⁡(a/d).     (A.6)
 MathType@MTEF@5@5@+=feaafiart1ev1aaatCvAUfKttLearuWrP9MDH5MBPbIqV92AaeXatLxBI9gBaebbnrfifHhDYfgasaacH8akY=wiFfYdH8Gipec8Eeeu0xXdbba9frFj0=OqFfea0dXdd9vqai=hGuQ8kuc9pgc9s8qqaq=dirpe0xb9q8qiLsFr0=vr0=vr0dc8meaabaqaciaacaGaaeqabaqabeGadaaakeaafaqabeqadaaabaGaem4qam0aaSbaaSqaaiabigdaXiabc6caUiabc6caUiabiAda2aqabaGccqGH9aqpdaWcaaqaaiabgkHiTiabikdaYiabdAfawnaaBaaaleaacqaIWaamaeqaaaGcbaGaeGOnayJagiiBaWMaei4Ba8Maei4zaC2aaeWaaeaacqWGHbqydaahaaWcbeqaaiabiwda1aaakiabc+caViabiAda2iabdsgaKnaaCaaaleqabaGaeGynaudaaaGccaGLOaGaayzkaaGaeyOeI0IaeGymaeJaeGOmaiJagiiBaWMaei4Ba8Maei4zaC2aaeWaaeaacqWGHbqycqGGVaWlcqWGKbazaiaawIcacaGLPaaaaaGaeiilaWcabaGaem4qam0aaSbaaSqaaiabiEda3aqabaGccqGH9aqpcqGHsislcqaI2aGncqWGdbWqdaWgaaWcbaGaeGymaeJaeiOla4IaeiOla4IaeGOnaydabeaakiabcYcaSaqaaiabdoeadnaaBaaaleaacqaIWaamaeqaaOGaeyypa0JaemOvay1aaSbaaSqaaiabicdaWaqabaGccqGHsislcqaI2aGncqWGdbWqdaWgaaWcbaGaeGymaeJaeiOla4IaeiOla4IaeGOnaydabeaakiGbcYgaSjabc+gaVjabcEgaNnaabmaabaGaemyyaeMaei4la8IaemizaqgacaGLOaGaayzkaaGaeiOla4caaaaa@72D5@

## Competing interests

The author(s) declare that they have no competing interests.

## Authors' contributions

All authors contributed equally to this work

All authors read and approved the final manuscript.
